# Population pharmacokinetics of meropenem-vaborbactam in acutely ill hospitalized patients with various degrees of renal dysfunction including continuous renal replacement therapy

**DOI:** 10.1128/aac.00108-25

**Published:** 2025-06-02

**Authors:** Griffin Reed, Adrian Valadez, Brandon J. Smith, Erin K. McCreary, Ellen G. Kline, MIchael N. Neely, Kevin M. Squires, Ryan K. Shields, Nathaniel J. Rhodes

**Affiliations:** 1 Department of Pharmacy Practice, College of Pharmacy, Midwestern University 15475, Downers Grove, Illinois, USA; 2 Pharmacometrics Center of Excellence, Midwestern University 69281, Downers Grove, Illinois, USA; 3 University of Pittsburgh, Division of Infectious Diseases 271847, Pittsburgh, Pennsylvania, USA; 4 Keck School of Medicine, University of Southern California 12223, Los Angeles, California, USA; 5 Laboratory of Applied Pharmacokinetics and Bioinformatics, The Saban Research Institute, Children’s Hospital of Los Angeles 466592, Los Angeles, California, USA; 6 University of Pittsburgh, School of Medicine 12317, Pittsburgh, Pennsylvania, USA; 7 Department of Pharmacy, Northwestern Memorial Hospital 24560, Chicago, Illinois, USA; Providence Portland Medical Center, Portland, Oregon, USA

**Keywords:** beta-lactamases, cumulative fraction of response, *Klebsiella pneumoniae*, PK/PD

## Abstract

Real-world meropenem-vaborbactam (MVB) pharmacokinetic-pharmacodynamic (PK/PD) attainment data in patients with renal dysfunction including continuous renal replacement (CRRT) are lacking. We evaluated MVB PK in patients with renal dysfunction and CRRT. Patients requiring hemodialysis were excluded. Plasma concentrations were quantified using LC-MS/MS. Multiple compartmental PK models and covariate effects were evaluated using Pmetrics 2.1.1. Free (f) fractions of 98% and 67% were assumed with targets of 40%– 100% *f*T_>MIC_ and *f*AUC/MIC >38 for meropenem (MEM) and vaborbactam (VAB), respectively. Individual patient PK/PD attainment was evaluated using Bayesian posterior predictions. Probability of target attainment (PTA) stratified by MIC and cumulative fraction of response (CFR) vs the EUCAST *K. pneumoniae* distribution were evaluated for 2 and 4 g MVB regimens. Eighteen patients (54% female, 17% CRRT) aged 54 ± 14 years, weighing 91 ± 31 kg with a CrCL of 114 ± 102 mL/min/1.73 m^2^ at baseline, contributed 83 plasma samples. For each drug, a one-compartment PK model was identified. Non-CRRT and residual clearance of MEM was higher than VAB, resulting in VAB accumulation. Individual-patient target attainment was variable. MEM PTA was >90% at MICs up to 0.5 mg/L, and VAB PTA was >90% at MICs up to 4 mg/L across renal states. CFR for MEM was >80%, and CFR for VAB was >70% in patients with renal dysfunction or CRRT and standard PK/PD targets with renal dosing regimens. For infections with MICs up to 1 mg/L, MVB regimens of 2 g IV every 8 h were adequate in CRRT. Studies linking MVB attainment to clinical outcomes are needed.

## INTRODUCTION

Patients infected with carbapenem-resistant Enterobacterales (CRE) including *Klebsiella pneumoniae* have high rates of mortality and treatment failure when treated with sub-optimal antimicrobials. Meropenem-vaborbactam (MVB), a beta-lactam-beta-lactamase inhibitor combination (1), was developed to address the need to restore carbapenem activity against *Klebsiella pneumoniae* carbapenemase (KPCs)-producing Enterobacterales. MVB is administered in a 1:1 ratio over 3 h to maximize the pharmacokinetic-pharmacodynamic (PK/PD) target attainment for the combination of the two agents and suppress the emergence of resistance (1, 2). Population PK studies supporting MVB development did not include critically ill patients on continuous renal replacement therapy (CRRT) (3, 4); thus, the evidence related to optimal dosing in CRRT is limited to case reports and *ex vivo* models (5, 6).

We sought to evaluate the PK of MVB in patients with various degrees of renal insufficiency, including those requiring CRRT. Prior research evaluating beta-lactam-beta-lactamase inhibitors suggests that receipt of CRRT is a risk factor for treatment failure (7, 8). The minimum PK/PD target for MEM is 40% *f*T_>MIC_; however, current guidelines suggest at least 100% *f*T_>MIC_ as a preferred target to improve clinical outcomes, especially in critically ill patients (9). In this study, we aimed to evaluate individual and population PK/PD target attainment in critically ill patients treated with MVB, including those requiring CRRT.

## MATERIALS AND METHODS

### Design and patients

Patients receiving MVB at the University of Pittsburgh Medical Center from December 2017 to July 2022 were approached to enroll in a pharmacokinetic sampling study. MVB was used for suspected or confirmed CRE infections per standard care and in accordance with institutional guidance, as previously reported (10). Eligible patients had to be treated with at least 48 h of MVB prior to PK sampling. The 48 h threshold was chosen to approximate the steady state in the population. The protocol was approved by the University of Pittsburgh Institutional Review Board (protocol # STUDY19040041). Informed consent was obtained from individual patients or their legally authorized representatives. Covariate data, including demographic (age and sex), morphometric (weight and BSA), and clinical (serum creatinine, CRRT status, and dosing records) parameters were obtained from electronic health records (Cerner, Oracle Corporation) by trained personnel. Patients were excluded from this analysis if they required concurrent hemodialysis, peritoneal, or low-efficiency dialysis. Creatinine clearance (CrCL) was estimated using the Cockcroft-Gault method (11) and normalized to a body surface area of 1.73 m² for standardized comparisons across patients with differing body compositions. The study leveraged a prospective sampling protocol to capture pharmacokinetic variability across a heterogeneous, real-world cohort. De-identified data analysis was conducted at Midwestern University (determined to be non-human subjects research by the Midwestern University IRB).

### Dosing and sample collection

MVB dosing was guided by institutional renal dosing protocols. Blood samples were collected at the following time points per protocol: pre-dose, and 1.5, 3, 4, 6, 8, and 12 h post-dose, when feasible and applicable. For patients unable to complete full sampling, a minimum of two samples were collected at least 3 h apart. Pre-filter blood samples were collected in patients requiring CRRT. Approximately 3 mL of blood was collected at each time point using EDTA tubes (BD Vacutainer), placed at 4°C within 15 min of collection for up to 12 h after the initial blood collection. Plasma was separated by centrifugation, then aliquoted and stored at −80°C prior to analysis.

### LC-MS/MS assay

Following Food and Drug Administration (FDA) guidance (12) and a previously published method (13), we measured VAB and MEM on a Shimadzu NexeraXD UHPLC with a Shimadzu 8045 triple quadrupole mass spectrometer (Shimadzu Scientific Instruments, Columbia, MD). The assay was validated internally, demonstrating reproducibility and linearity over a range of 0.1–100 µg/mL for both compounds. Total concentrations were determined following protein precipitation with acetonitrile. Mobile phases A and B were 1 mM formic acid in water and acetonitrile (Fisher Scientific, Hampton NH), respectively. An elution gradient was run using an Agilent Poroshell HPH C18 Atlantis T3 column (2.7 µm, 50 × 2.1 mm; with guard). Sulbactam (Sigma Aldrich, St. Louis, MO) and meropenem-d6 (Toronto Research Chemical, Toronto Canada) were used as internal standards for VAB and MEM, respectively. Transitions for VAB (MedChemExpress, Monmouth Junction, NJ) were 296.15 → 234.15 and 278.15, and for MEM (USP, Rockville, MD), they were 384.10 → 68.05, 141.1, and 114.05.

### Model development

Pharmacokinetic modeling was performed using the NPAG algorithm available in the *Pmetrics* package (version 2.1.1) for R (version 4.1.2) (14, 15). Multiple compartmental models were considered. Both one-compartment and two-compartment models were evaluated. Covariate effects were evaluated in subsequent models. Independent effects of CRCL and CRRT on CL were evaluated *a priori*. Improvements in goodness-of-fit, log-likelihood, bias/imprecision, and Akaike Information Criterion (AIC) were used to select the final model. The system was described using an inhomogeneous set of differential equations structured as follows:


$$1.\;dX[1]=Rate_{in(drug)}-(K_{12}+K_{10})\times X[1]+K_{31}\times X[3]$$

$$2.\;dX[2]=K_{12}\times X[1]-(K_{23}+FLOW\times K_{20})\times X[2]$$

$$3.\;dX[3]=K_{23}\times X[2]-K_{31}\times X[3]$$

Where MEM and VAB were modeled in parallel as zero-order inputs to the system (Rate_in_). Transfer rate constants described the movement of both drugs from pre-filter blood to the CRRT filter (K_12_) and from the CRRT filter to post-filter blood (K_23_) then back to pre-filter blood (K_31_) when CRRT was active. If CRRT was not active, elimination occurred only via K;_10_ otherwise, elimination via K_20_ was also possible. The effluent flow rate (i.e., the sum of ultrafiltration and dialysate flow rates) was incorporated into the model using the term FLOW, which was standardized to the median flow rate (mL/h).

### Covariate models

We evaluated the impact of patient-specific covariates using regressions of the observed covariate vs. PK parameter relationships for continuous variables (e.g., CrCL and total body weight) or boxplots for categorical variables (e.g., sex, AKI, CRRT). Both linear and non-linear relationships between PK parameters and continuous covariates were evaluated.

### Individual PK/PD target attainment

Individual PK/PD target attainment rates were evaluated using Bayesian posterior predictions. Attainment was assessed using the EUCAST MIC_50_ (0.75 mg/L) as a representative susceptibility threshold for *K. pneumoniae* (Fig. S3A) but is still above a clinically relevant modal MIC for KPC-producing organisms based on epidemiology data within our center (Fig. S3B). Additional analysis using a breakpoint MIC of 4 mg/L was conducted to reflect less susceptible isolates that may be encountered in clinical practice. For each patient, we calculated the percent of the dosing interval during which unbound concentrations remained above the MIC over the first 24 h of treatment (*f*T_>MIC_) for MEM and the ratio of the unbound area under the concentration-time curve relative to the MIC (*f*AUC/MIC) for VAB. Free concentrations were estimated using published protein binding constants for MEM and VAB (i.e., 1-protein binding) (1). We evaluated two MEM PK/PD targets: 100% *f*T_>1xMIC_ and 100% *f*T_>4xMIC_. These targets were applied against the EUCAST MIC_50_ of *K. pneumoniae* (0.75 mg/L). For VAB, the PK/PD target evaluated was *f*AUC/MIC >38 (2, 16). The results were stratified according to CRRT status.

### Monte Carlo simulations

Monte Carlo simulations were conducted using the final covariate-adjusted compartmental PK model using the *Pmetrics* package for R (14). Simulations assessed the probability of target attainment (PTA) at a fixed MIC value of 0.03 (i.e., the median UPMC *K. pneumoniae* MIC) to up to 16 mg/L (i.e., the 2024 CLSI resistant breakpoint), as well as the cumulative fraction of response (CFR) vs. the EUCAST *K. pneumoniae* MIC distribution (17). The latter analysis served as a worst-case scenario for empiric treatment, whereas the former scenario reflects the treatment of susceptible CRE infections. Supplemental analyses using an MIC distribution from the University of Pittsburgh Medical Center (UPMC) (Fig. S3) were performed concurrently to reflect local epidemiology and provide clinically relevant CFR estimates (Fig. S4). The UPMC distribution was derived from previously published local data (18), enriched primarily for KPC-producing isolates. Simulations evaluated PK/PD targets of 40% *f*T_>MIC_ and 100% *f*T_>MIC_ for MEM and *f*AUC/MIC >38. We simulated the following regimens: 2 g (1 g meropenem/1 g vaborbactam) or 4 g (2 g/2 g) IV administered every 8 h over 3 h. For each regimen, 1,000 simulated profiles were generated using semi-parametric sampling from the final model (19). Predictions were generated every 0.2 h for the first 24 h of dosing. In all simulations, body weight was held constant to focus on the impact of renal status. Effluent flow was fixed at 1750 mL/h (25 mL/kg/h for a 70 kg patient) for CRRT. Renal function was evaluated at CrCL values of 50 or 120 mL/min, standardized to 1.73 m² BSA.

### Safety evaluation

We evaluated the safety of individual patients and simulated MEM and VAB dosing regimens using the upper bound 90th percentile of *f*AUC_0-24hr_ for each drug (i.e., >1306.34 34 mg*h/L and >1373.5 mg*h/L, respectively) based on exposures from phase 1 and 3 cUTI studies (20).

## RESULTS

### Patient characteristics

In this cohort, 18 patients (44% male) provided a total of 83 plasma samples for analysis. Table 1 summarizes the demographic and clinical characteristics of these patients. The mean ± SD age was 54.7 ± 14.4 years, and the baseline CrCL was 123.3 ± 103.7 mL/min/1.73 m^2^. There were three (16.7%) patients who required CRRT. Among these, the median effluent flow rate was 2,000 mL/h, the median blood flow rate was 250 mL/min, the median dialysate flow rate was 1500 mL/h, and the median pre- and post-replacement flow rate was 250 mL/h. Figure S1 shows the raw observed MEM and VAB concentrations relative to the last observed dose.

**TABLE 1:** Demographic data and clinical characteristics^*a*^

Demographics (N=18)	Measurement^*b*^
Age (y)	54.7 ± 14.4
TBW (kg)	90.7 ± 32.6
BSA (m*^2^*)	2.02 ± 0.36
SCr (mg/dL)	0.99 ± 0.74
Baseline CrCL (mL/min/1.73 m^2^)	123.3 ± 103.7
AKI not on CRRT, n (%)	2 (11.1%)
CRRT, n (%)	3 (16.7%)
Sex, n (%)	
Male	8 (44%)
Female	10 (56%)

^*a*^ Acute Kidney Injury (AKI), Body Surface Area (BSA), Creatinine Clearance (CrCL), Continuous Renal Replacement Therapy (CRRT), Serum Creatinine (SCr), Total Body Weight (TBW).

^*b*^ Measurement is mean ± SD unless otherwise specified.

### Model development

**Fig 1:**
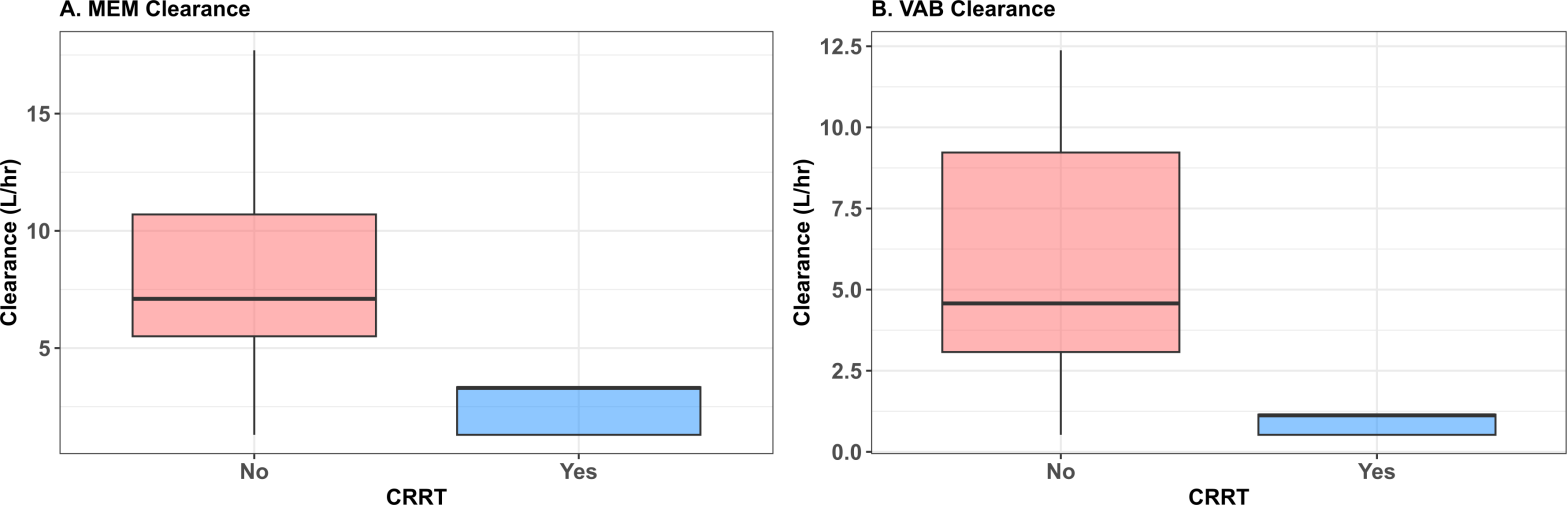
Differences in MEM (**A**) and VAB clearance (CL) (**B**) between patients with and without continuous renal replacement. (**A**) MEM, meropenem CL and (**B**) VAB, vaborbactam CL stratified by CRRT status (Yes/No). Boxplots display the median and interquartile range for the Bayesian posterior distribution based on covariate values across all time points stratified by CRRT status.

Table S1 provides a detailed comparison of the model build. The base three-compartment model (pre-filter central compartment, filter compartment, and post-filter compartment) included the effects of CRRT and effluent flow rate on drug elimination and yielded an OFV of 975.8 (model 1). A four-compartment model (model 2) with an added peripheral compartment resulted in increased −2*LL and AICs and was rejected. Parameterization of the base three-compartment model with different baseline clearances for CRRT and non-CRRT patients yielded a significant improvement (model 3 vs. model 1, ΔOFV = 9.2). Parameterization of WT (kg) on volume (model 4) also yielded a significant improvement (model 4 vs. model 1, ΔOFV = 10.6). Parameterization of CrCL (mL/min/1.73 m^2^) on non-CRRT clearance also yielded a significant improvement (model 5 vs. model 1, ΔOFV = 8.3). Regression analyses comparing unadjusted versus BSA-normalized CrCL in non-CRRT patients demonstrated that the BSA-normalized approach provided a superior fit, with an AIC reduction from 7492.841 to 7288.027 and improved covariate-parameter diagnostics, supporting its use in the final model. No other significant covariate effects were identified. As model 4 (CrCL on CL) and model 5 (WT on V) both significantly improved fitness, the combination of these effects was also evaluated (model 6 vs. model 1, ΔOFV = 8.6). Model 6 had the lowest population bias and was selected as the final model (Table S1). Figure 1 shows the distributions for total CL of MEM and VAB based on the Bayesian posterior distributions for patients CRRT and non-CRRT patients across all time points.

**Fig 2:**
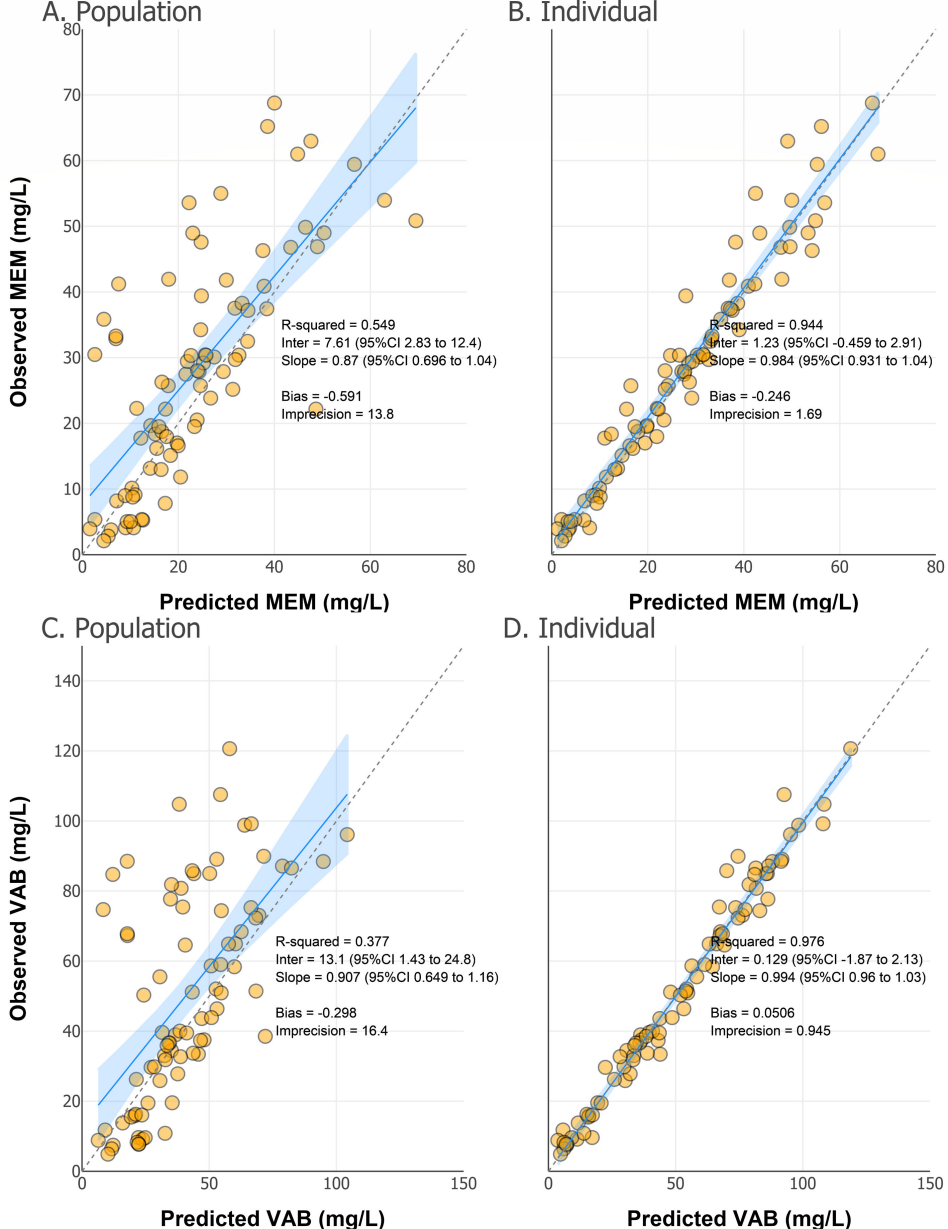
Population and individual-predicted concentrations versus observed concentrations from the final covariate-adjusted model of meropenem and vaborbactam. Gold circles represent individual data points, and the solid line indicates the line of unity (perfect prediction). The shaded blue area denotes the 95% CI for the model’s predictive performance. Metrics quantifying model fit and accuracy included R^2^, slope, intercept, bias, and imprecision for population (**A and C**) and individual (**B and D**) predictions.

The goodness-of-fit plots for population and individual-predicted versus observed meropenem and vaborbactam concentrations are shown in Fig. 2. The population model for meropenem demonstrated an R^2^ of 0.549 with a slope near unity and low bias (−0.591), whereas the individual-predicted model improved predictive accuracy (R^2^ = 0.944, bias = −0.246. For VAB, population predictions had a lower R^2^ of 0.377, although individual predictions improved substantially (R^2^ = 0.976). Bias and imprecision were within acceptable limits for both drugs, supporting the model’s predictive performance. Of note, the final model parameters had shrinkage <30%, indicating informative sampling and reasonable fits for each parameter. Overall, the final two-input, two-output, one-compartment population PK model was deemed appropriate for capturing MVB PK in this critically ill population. Table 2 summarizes the population PK parameters of the final model. Equations for the final covariate-adjusted model are shown in Equations 1 and 2:


(1) $$CL=CL_{nonCRRT}\times \left(\frac{CrCL}{120}\right)\times \left(1-CRRT\right)+CL_{CRRT}\times (CRRT)$$

**TABLE 2:** Median parameters and 95% credible intervals for meropenem and vaborbactam^*a*^

Parameter	Median (weighted)	95% Credible interval	Shrinkage (%)
Meropenem			
CL_non-CRRT_ (L/h):	10.6	9.13–12.5	10.4%
CL_residual_ (L/h):	2.1	1.24–2.85	19.2%
K_12_ (h^−1^)	0.77	0.43–1.16	10.9%
K_20_ (h^−1^)	4.44	2.21–5.50	11.8%
K_23_ (h^−1^)	29.3	20.6–31.9	9.9%
K_31_ (h^−1^)	1.67	0.51–35.0	24.6%
Vd (L):	27.4	23.2–38.4	9.1%
Vaborbactam			
CL_non-CRRT_ (L/h):	6.64	3.99–7.84	10.2%
CL_residual_ (L/h):	1.15	0.90–1.42	12.0%
K_12_ (h^−1^)	1.45	0.97–2.70	20.0%
K_20_ (h^−1^)	0.59	0.18–0.66	13.5%
K_23_ (h^−1^)	22.2	20.3–26.2	22.0%
K_31_ (h^−1^)	4.31	2.56–30.7	27.7%
Vd (L):	24.3	19.7–31.1	9.1%

^*a*^ Median (weighted) population PK parameters and associated 95% credible intervals for MEM and VAB. Parameters include population typical values for creatinine clearance normalized clearance (CL_non-CRRT_ in L/h) and CL_residual_ on CRRT (L/h), weight normalized volume of distribution in L (V / 80 kg), the elimination rate constant of the filter (K_20_), and intercompartmental transfer rates (in h^−1^).


(2) $$Vd=V1\times \frac{WT}{80}$$

### Individual PK/PD target attainment

**Fig 3:**
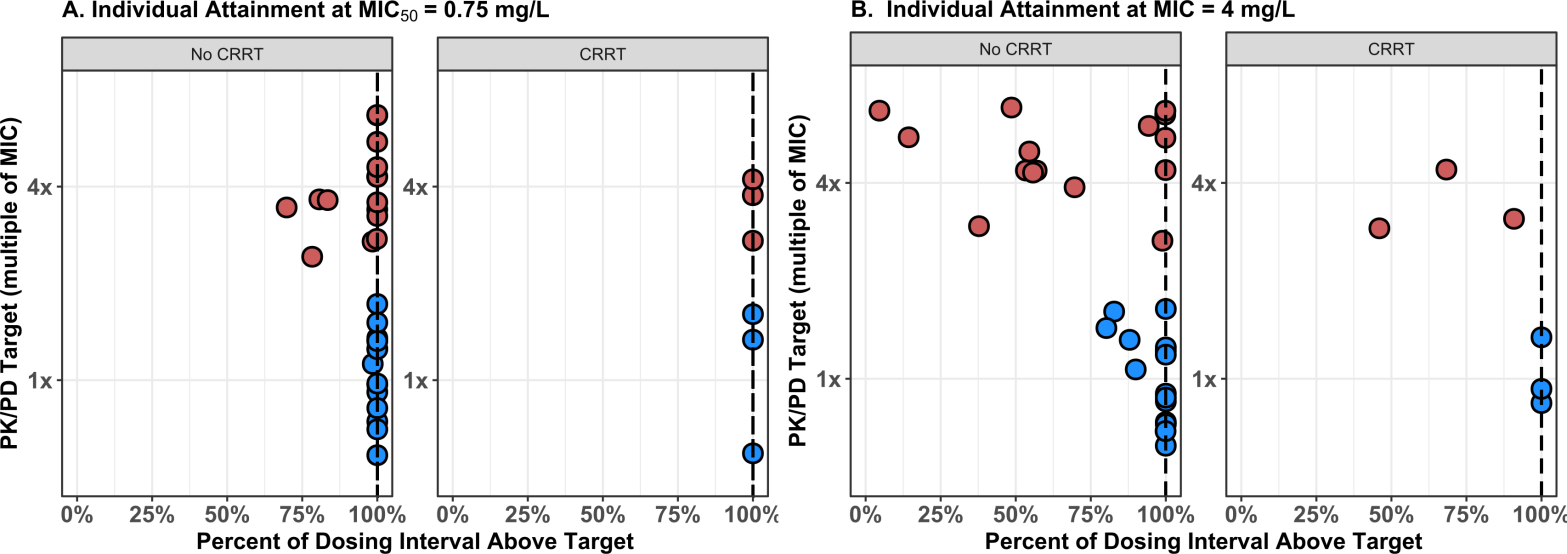
Individual target attainment 1x (blue) and 4x (red) MIC targets for *K. pneumoniae* MIC_50_ and breakpoint MIC. (A) Individual patient PK/PD target attainment for 100% *f*T_>MIC_ for meropenem at 1x and 4x the MIC_50_ for *K. pneumoniae* in the EUCAST MIC distribution stratified by CRRT status. (B) Individual patient PK/PD target attainment for 100% *f*T_>MIC_ for meropenem at 1 x and 4 x the breakpoint MIC of 4 mg/L for Enterobacterales stratified by CRRT status.

Individual target attainment rates for the *K. pneumoniae* MIC_50_ (MIC = 0.75 mg/L) are shown in Fig. 3 stratified by CRRT status. Panel A shows the results considering the MIC_50_ for *K. pneumoniae* in the EUCAST MIC distribution. A total of 14 of 15 (93%) non-CRRT patients and 3 of 3 (100%) CRRT patients achieved a PK/PD target of 100% *f*T_>MIC_ for meropenem at the 1xMIC goal. At the 4xMIC goal, attainment rates fell to 67% for non-CRRT but remained high for CRRT patients (100%). Similarly, at the breakpoint MIC of 4 mg/L, 73% of non-CRRT patients and 100% of CRRT patients achieved the 1xMIC goal. However, at the 4xMIC, the goal for the breakpoint MIC attainment rates fell to 26.7% for non-CRRT patients and 0% for CRRT patients. At both the MIC_50_ and the breakpoint MIC, individual patient *f*AUC/MIC >38 for VAB rates were high irrespective of renal replacement status (93%–100% vs. breakpoint MIC and 100% vs. MIC_50_). None of the patients experienced 24 h VAB *f*AUCs exceeding the safety threshold of 1373.5 mg*h/L. Similarly, no patient exhibited a 24 h MEM *f*AUC exceeding the safety threshold of 1306.34 mg*h/L.

### Monte Carlo simulations

**Fig 4:**
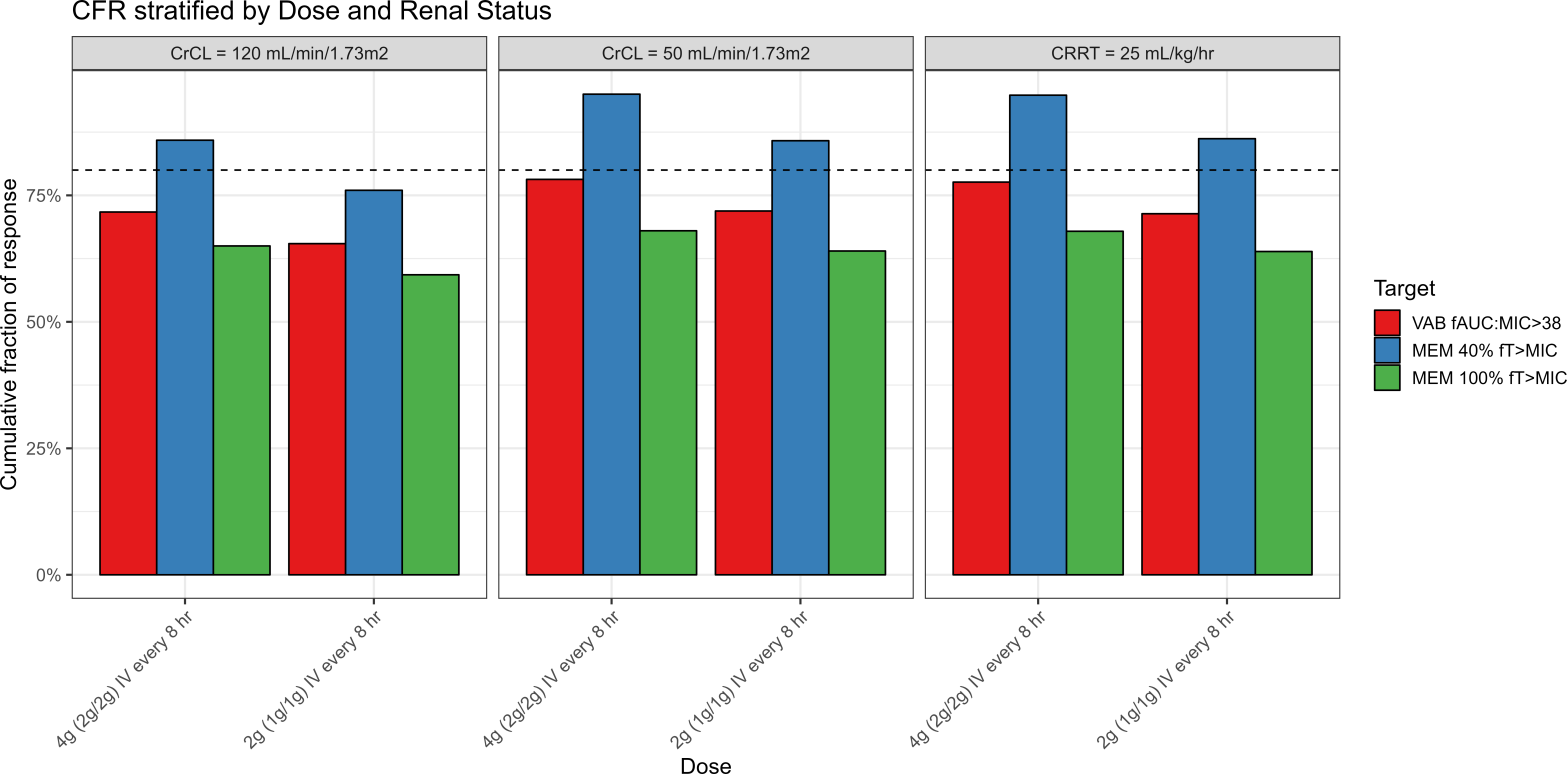
MV cumulative fraction of response vs. *K. pneumoniae* EUCAST MIC distribution. CFR values of MEM at 40% and 100% *f*T_>MIC_ for the EUCAST MIC distribution of *K. pneumoniae* stratified by CRRT status and dosing regimens. Red bars represent the fAUC/MIC target for VAB, blue bars represent the 40% *f*T_>MIC_ target for MEM, and green bars represent the 100% *f*T_>MIC_ target for MEM. The dashed line indicates the CFR target of 80%.

The results of the CFR analyses are shown in Fig. 4. MEM doses of 2 g IV every 8 h over 3 h yielded >80% CFR at the 40% *f*T_>MIC_ MEM target for patients with CrCL of 120 and 50 mL/min/1.73 m^2^, although 1 g IV every 8 h over 3 h was also adequate for patients with a CrCL of 50 mL/min/1.73 m^2^ at this target. CFR was reduced markedly for the 100% *f*T_>MIC_ MEM target ranging from 65% to 68% based on renal state (Fig. 4) with the 2 g MEM dose. VAB CFR ranged from 72% to 78% with the 2 g dose and from 66% to 72% with the 1 g dose based on renal state (Fig. 4). Simulated patients receiving the 1 g VAB dosing regimen did not exceed the upper bound *f*AUC safety margin. Among simulated patients receiving the 2 g regimen, 14.2% of CRRT and 30.5% of patients with a CrCL of 50 mL/min/1.73 m^2^ exceeded the upper bound VAB *f*AUC safety margin. In a sensitivity analysis using the UPMC MIC distribution for *K. pneumoniae*, CFR values were stratified by dosing regimen and renal status for both MEM (at 40% and 100% *f*T_>MIC_) and VAB (*f*AUC/MIC) (Fig. S4). Contrasting the primary analysis, regimens generally exceeded the 80% CFR threshold across the evaluated CrCL categories vs. the UPMC MIC distribution. Notably, MEM targeting 100% *f*T_>MIC_ maintained CFR values above 80% in patients with normal and reduced renal function. VAB also achieved high CFRs in most scenarios.

**Fig 5:**
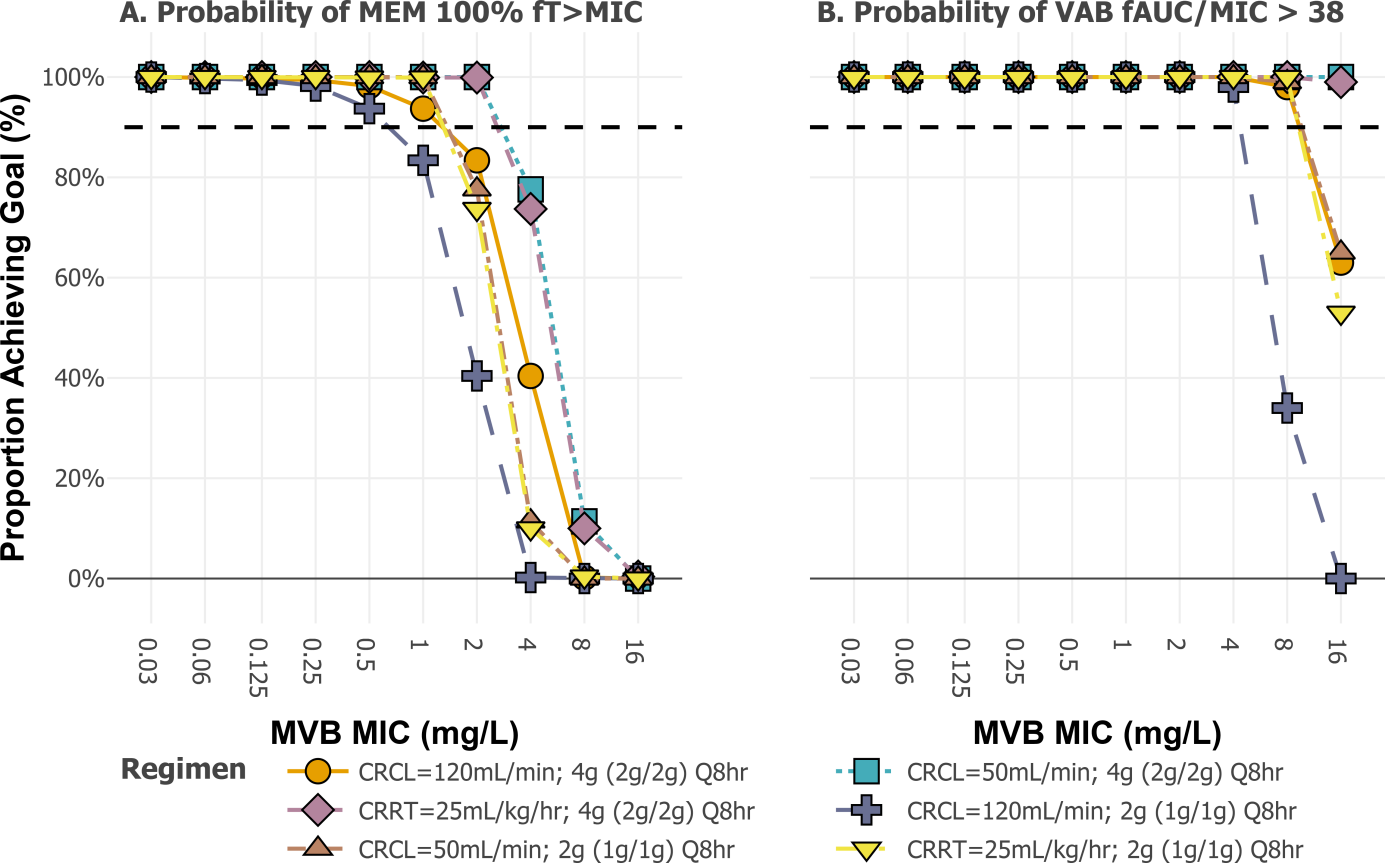
Probability of PK/PD target attainment for MEM (**A**) and VAB (**B**) stratified by MIC. CRCL, creatinine clearance; CRRT, continuous renal replacement therapy; and MVB MIC, meropenem vaborbactam MIC determined in the presence of fixed 8 mg/L vaborbactam.

Results of the PTA analysis for MEM at a goal of 100% *f*T_>MIC_ are shown in Fig. 5. For MEM, PK/PD attainment was >90% for MEM 2 g dose regimens at MICs up to 1 mg/L whereas patients with renal dysfunction or CRRT had >90% PTA with 2 g doses at MICs up to 2 mg/L. Similarly, 1 g dose regimens for renally impaired patients had >90% PTA at MICs up to 1 mg/L but fell below a 90% threshold at MICs of 2 mg/L or greater (Fig. 5). Patients with preserved renal function (e.g., CrCL = 120 mL/min/1.73 m^2^) had >90% PTA at MICs up to 1 mg/L with 2 g regimens and MIC up to 0.5 mg/L with 1 g dose regimens. On the other hand, PTA was >90% for VAB at 1 or 2 g doses for MICs up to 8 mg/L except for the 1 g IV every 8 h regimen at a CrCL of 120 mL/min/1.73 m^2^.

## DISCUSSION

We report the first population PK study of MEM and VAB wherein CRRT was explicitly modeled in acutely ill patients with CRE infections. Our PK model attempted to represent CRRT physiology wherein drug elimination via the circuit or residual clearance independent of CrCL, and this model adequately predicted MEM and VAB in plasma and was considered fit for purpose for predicting PK profiles at the individual patient level as well as the population level.

Compared to patients with normal renal function, we found that CRRT was associated with a 54% reduction in clearance, leading to a greater accumulation of vaborbactam relative to meropenem. These findings align with prior observations, including the *ex vivo* study by Sime et al., which evaluated the impact of CRRT settings and filter characteristics on the PK of meropenem and vaborbactam (6). The investigators found that MEM and VAB clearance in their CRRT model was approximately 1.9 L/h and 1.4 L/h, respectively, at a blood flow rate of 200 mL/min with AN69 ST100 filter types and pre-filter dilution. Among our CRRT patients, a balanced pre- and post-filter fluid replacement was used, and the median blood flow rate was 200 mL/min. Our population model estimates for CL_residual_ for MEM and VAB were 2.1 and 1.15 L/h, which approach the *ex vivo* model estimates. Likewise, our model parameters demonstrated relatively low non-parametric shrinkage and good posterior fits, indicating that the model fitting algorithm was able to identify sufficiently informative prior distributions for our patients. Notably, Sime et al. also highlighted the significant impact of filter surface area on drug clearance, with larger filters (1.5 m^2^ vs. 1.0 m^2^) achieving up to 50% higher sieving coefficients for meropenem (6). Although our study did not stratify clearance based on filter size, variability in drug clearance observed without our CRRT cohort may partially reflect such technical differences.

Limited clinical data exist evaluating vaborbactam elimination during continuous dialysis. In a clinical case report, Kufel et al. found that MEM and VAB, administered as 2 g (1 g / 1 g) every 8 h in a critically ill patient on continuous venovenous hemodialysis (CVVHD) over the course of 12 days, had mean extraction ratios of 47.97% and 41.24% and t_1/2_ of 6.38 hours and 16.81 h for MEM and VAB, respectively, when using a NxStage System One 1.6 m^2^ polyethersulphone membrane filter with a blood flow rate of 250 mL/min and an ultrafiltration flow rate of 3 L/h (5). Extraction ratios were measured as prefilter and postfilter concentrations, and no residual urine output was documented. These data indicated that MEM and VAB extraction via CVVHD differed, with the mean extraction of VAB being 14% lower relative to MEM, thus contributing further to lower VAB clearance in this population, and an overall VAB clearance rate which was approximately 38% that of MEM. Data from patients undergoing hemodialysis showed that vaborbactam HD clearance was 40% lower than MEM HD clearance (21). We found that the elimination rate constant of the CRRT filter was roughly 8-fold lower for VAB compared with MEM in our population model; however, in CRRT, drug removal is affected by multiple factors including pre- and post-dilution not used in HD. The CL_residual_ estimate for MEM in the current study was 2.1 L/h, which approximates the CL_residual_ previously observed in HD patients (21). However, the CL_residual_ estimate for VAB in the current study is higher than that observed in HD patients (1.15 vs. 0.08 L/h) (21), which may be due to differences in the degree of renal impairment between CRRT and HD patients. Notably, we excluded HD patients given our study design; however, similar to the observations in HD, we also found that vaborbactam accumulates in CRRT.

Accumulation of vaborbactam in the setting of renal dysfunction and CRRT could hypothetically confer a protective effect on MEM in the setting of KPC infections. We conducted Monte Carlo simulations using our final population model and found that VAB CFR with the standard PK/PD target of *f*AUC/MIC >38 was <80% irrespective of renal state. Likewise, the attainment of 100% *f*T_>MIC_ for MEM was <80% for all evaluated doses. Notably, CFR represents a worst-case scenario as the EUCAST MIC distribution is enriched for resistant isolates, including metallo-beta-lactamase producers. However, genotypic resistance testing is not universal; thus, CFR for this distribution is salient when the genotype is unknown. The difficulty of achieving 4xMIC targets with MVB against organisms exhibiting elevated MICs at the highest labeled dose suggests a potential role for dose individualization (e.g., therapeutic drug monitoring). The CFR analysis demonstrated difficulties with attaining standard PK/PD targets across a published MIC distribution. PTA analysis across a clinically relevant range of MICs found that target attainment was >90% with MEM 1 or 2 g doses at MICs up to 1 mg/L and >90% with VAB 1 or 2 g doses at MICs up to 8 mg/L in patients with renal dysfunction and those requiring CRRT. Joint attainment of MEM and VAB targets was entirely driven by MEM up to an MIC of 4 mg/L, above which joint target attainment began to decline; this is reflected in the isometric pattern observed (Fig. 5), where nearly all patients achieved VAB targets across the MIC range. Supplemental analyses using the Pittsburgh-specific MIC distribution demonstrated improved CFR for meropenem and vaborbactam at both 40% and 100% *f*T_>MIC_ targets (Fig. S4).

We found that within our patient cohort, target attainment rates for a goal of 100% *f*T_>MIC_ for MEM were >93% vs. the *K. pneumoniae* MIC_50_ and >73% vs. a breakpoint MIC of 4 mg/L. Individual VAB attainment rates for a goal of *f*AUC/MIC >38 were >93% at both the MIC_50_ and breakpoint MIC across renal states. None of the individual patient exposures exceeded the upper bound *f*AUC margin based on FDA safety margins (20). Results of the VAB Monte Carlo simulations suggest that 1 g doses (i.e., MVB dose of 2 g) administered IV every 8 h over 3 h are likely to be sufficient and safe vs. susceptible isolates in the setting of CRRT.

Our study has strengths and limitations. A notable strength was the design wherein real-world patients with KPC infections underwent relatively rich PK sampling. One limitation of our study is that this was a single-center cohort with a relatively small number of CRRT patients. The small sample size, for example, three CRRT patients, limits generalizability; however, our study provides valuable real-world data that can inform dosing in this population. However, our study represents the largest population PK analysis of MVB in CRRT patients to date. Another limitation is the use of the Cockcroft-Gault equation as a population estimate for renal function. Although we recognize the limitations of the equation, it remains the most used method in clinical practice worldwide and therefore maintains translational relevance. Additionally, we are unable to establish a connection between PK/PD targets and clinical outcomes until larger sample sizes are available. In distinction to prior research evaluating MVB PK/PD for a MEM goal of 40% *f*T_>MIC_, we evaluated more aggressive targets in our simulation, reflecting current guidance for critically ill patients. Such aggressive targets (i.e., 100% *f*T_>MIC_) may not be necessary in less severely ill patients. CFR estimates based on the EUCAST MIC distribution represent a worst-case scenario that may overestimate resistance and underestimate CFR, whereas supplemental analyses with the UPMC MIC distribution may better reflect clinical practice. A given clinical scenario may lie between these two examples. Only total concentrations were available, so fixed free fractions for meropenem (98%) and vaborbactam (67%) were assumed, which may not fully capture alterations in protein binding in critically ill patients. Additionally, our simulations were limited to 3 h infusions without exploring extended or continuous infusion strategies. Future studies in alternative dosing strategies are needed.

### Conclusion

We identified a population PK model predicting both MEM and VAB PK in patients with various degrees of renal impairment, including those requiring CRRT. MEM had greater residual clearance than VAB in CRRT, resulting in VAB accumulation. At the individual patient level, target attainment rates were high vs. standard PK/PD targets for both MEM and VAB and resulted in safe exposures with renally adjusted doses. CFR analysis suggested that 100% *f*T_>MIC_ for MEM and *f*AUC/MIC >38 may be difficult to achieve against organisms with elevated MVB MICs. PTA analysis suggested that MVB doses of 4 g IV every 8 h should be adequate for MICs up to 1 mg/L for MEM mg/L across renal states, whereas coverage for VAB included MICs up to 8 mg/L. For infections with MICs up to 1 mg/L, MVB regimens of 2 g IV every 8 h in CRRT appear adequate and safe.

## SUPPLEMENTAL MATERIAL

Supplemental material aac.00108-25-s0001.pdf Table S1; Fig. S1 to S4.

ASM does not own the copyrights to Supplemental Material that may be linked to, or accessed through, an article. The authors have granted ASM a non-exclusive, world-wide license to publish the Supplemental Material files. Please contact the corresponding author directly for reuse.
