# Supplementary material for: Population pharmacokinetics of meropenem-vaborbactam in acutely ill hospitalized patients with various degrees of renal dysfunction including continuous renal replacement therapy
Source: Antimicrob Agents Chemother. 2025 Jun 2;69(7):e00108-25. doi: 10.1128/aac.00108-25 (PMC12217465; doi:10.1128/aac.00108-25)
Supplement: Supplemental material — Table S1; Fig. S1 to S4. [file aac.00108-25-s0001.pdf]

**TABLE S1:** Compare base models:

| *Model* | *Structure* | *Covariates* | *-2*LL* | *AIC* | *MEM Bias / VAB Bias* | *Comment* |
|---|---|---|---|---|---|---|
| *1* | 3-compartment base | — | 975.8 | 1004.2 | 3.3 / 2.34 | Base model |
| *2* | 4-compartment base | — | 1057.7 | 1095.2 | -3.5 / -8.3 | Higher AIC; rejected model |
| *3* | Model 1 with CL separated by CRRT | CRRT | 966.6 | 999.8 | 1.7 / 1.6 | Lower AIC and lower bias vs 1 |
| *4* | Model 3 with WT/80 kg on V | CRRT, WT | 965.2 | 998.4 | 3.1 / 3.4 | Lower AIC and higher bias vs 1 |
| *5* | Model 3 with CrCL/120 mL/min/1.73m$^2$ on CL | CRRT, CrCL | 967.5 | 1000.7 | -0.54 / 0.27 | Lower AIC and lower bias vs 1 |
| *6* | Models 3, 4 and 5 | CRRT, CrCL, WT | 967.2 | 1000.4 | -0.59 / -0.3 | Lower AIC vs 5, low bias; Final |

**Table legend:** The table outlines the progression of model-building steps, detailing the structural modifications, covariates incorporated, and their impact on model fit as assessed by -2*LL, bias, and AIC. Decisions to accept or reject models were based on improvements in statistical metrics, minimization of population bias and physiological relevance.

**Figure S1.** Spaghetti plot of observed MV plasma concentrations in n=18 adult patients

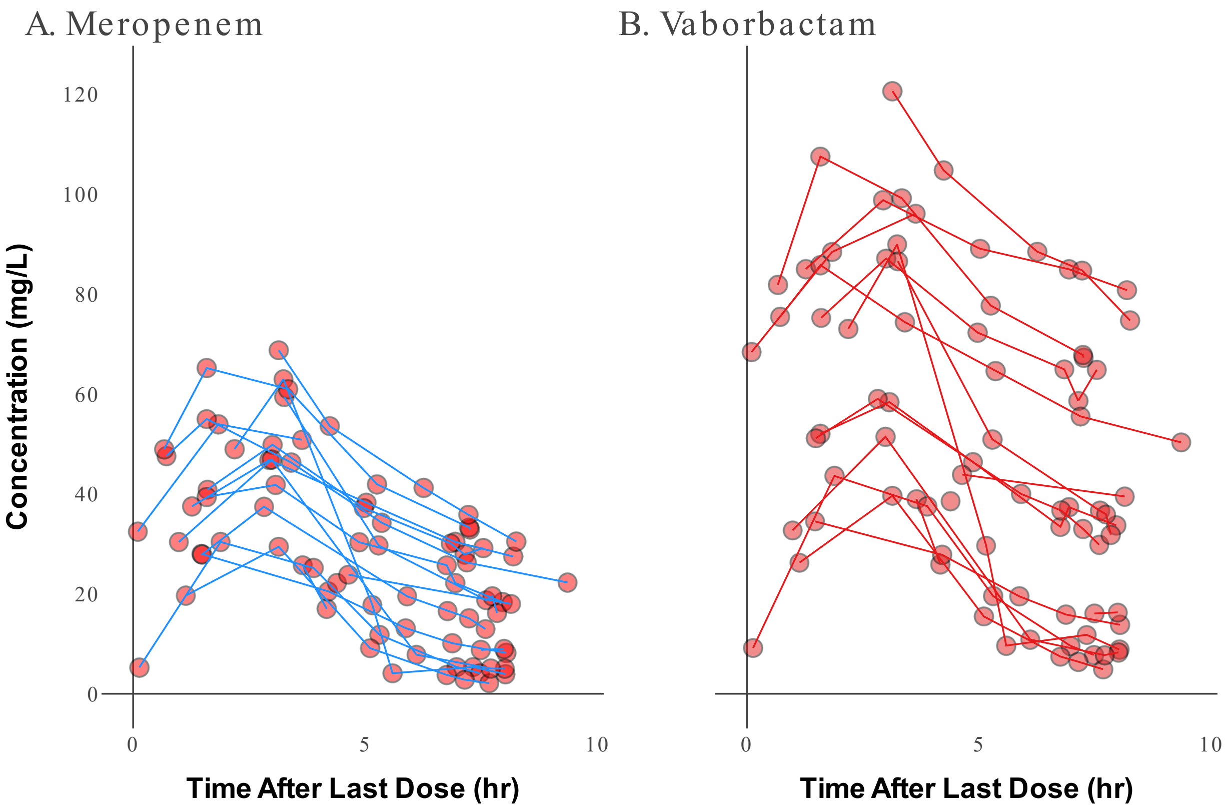


**Figure legend:** Lines represent concentrations from the same patient over time (after last dose).

**Figure S2.** Spaghetti plot of observed MV plasma concentrations in CRRT patients n = 3

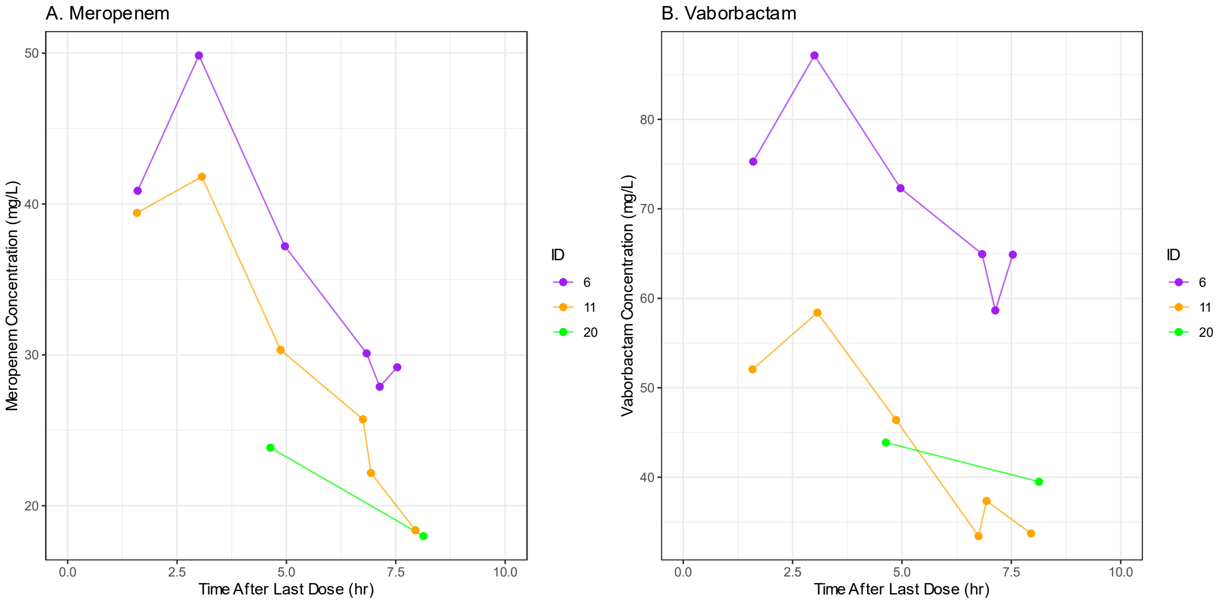


**Figure legend:** Lines represent concentrations from the same patient over time (after last dose). CRRT patients are represented by their respective ID's.

**Figure S3.** Comparison of MIC Distributions: EUCAST vs. UPMC Populations

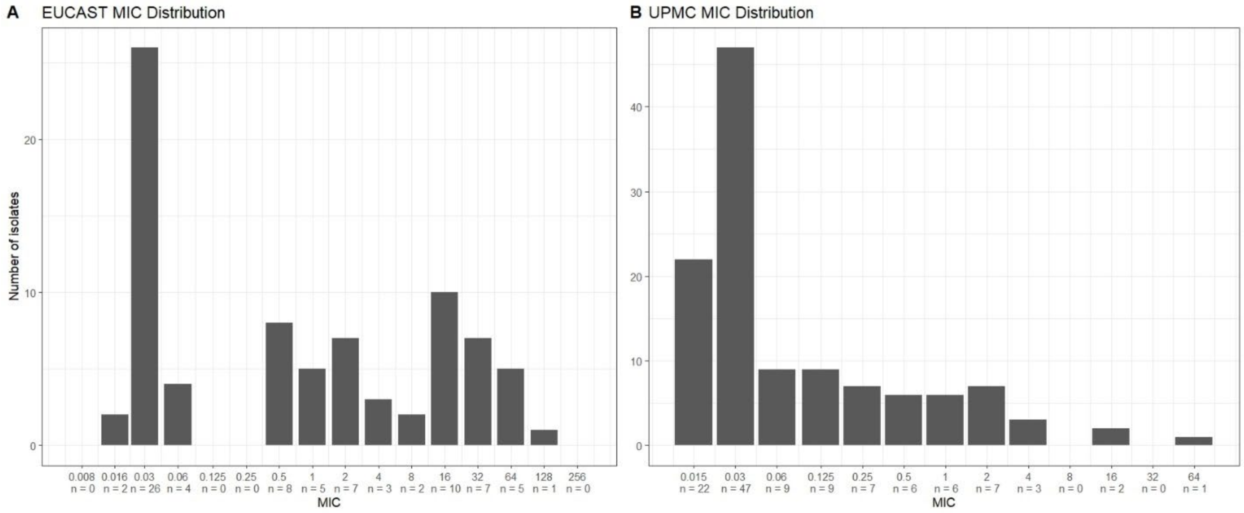


**Figure legend:** Histogram illustrates the distribution of MIC's among tested isolated, with the x-axis denoting MIC values and the y-axis representing density. Panel A. shows the EUCAST MIC distribution, and Panel B presents the UPMC MIC distribution.

**Figure S4.** Cumulative Fraction of Response (CFR) Against *K. pneumoniae* Pittsburgh MIC Distribution

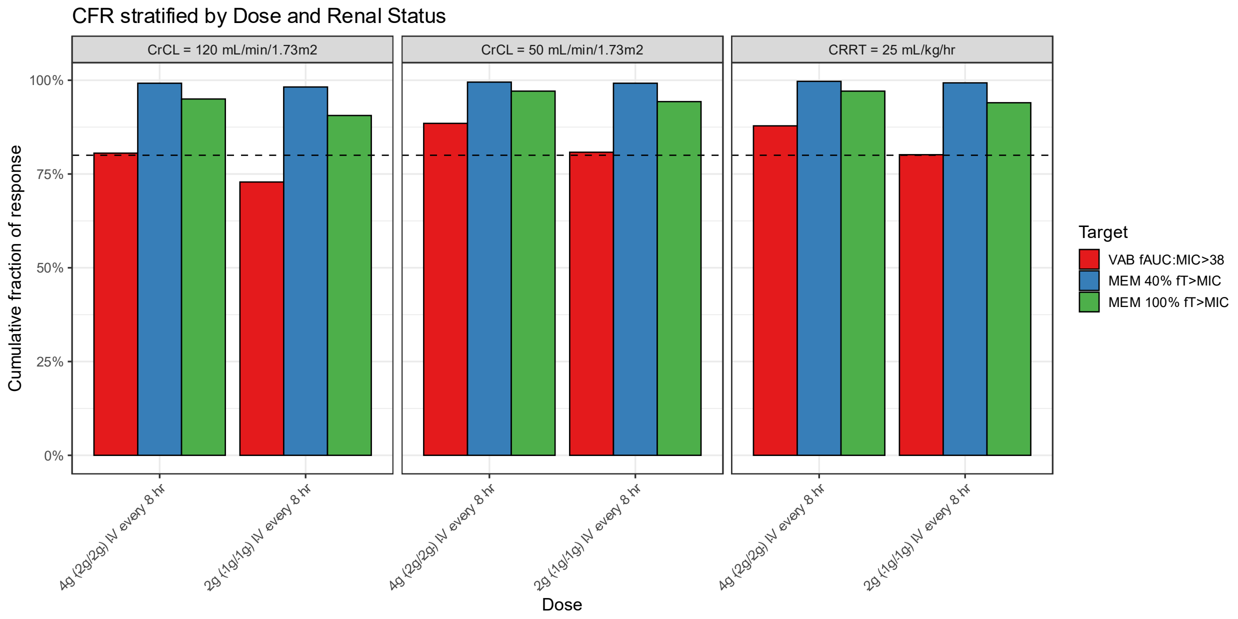


**Figure legend:** CFR values of MEM at 40% and 100% fT>MIC for the Pittsburgh MIC distribution of *K. pneumoniae*, stratified by CRRT status and dosing regimens. Red bars represent the fAUC/MIC target for VAB, blue bard represent the 40% fT>MIC targets for MEM, and green bard represent the 100% fT>MIC target for MEM. The dashed line indicated the CFR targets for 80%.
